# Editorial: Microbial symbiosis and infectious disease dynamics in reptiles and wildfowl

**DOI:** 10.3389/fmicb.2025.1673344

**Published:** 2025-08-13

**Authors:** Xiaoliang Hu, Hai Li, Aijing Liu, Zhenyu Zhang

**Affiliations:** ^1^Faculty of Agriculture, Forestry and Food Engineering, Yibin University, Yibin, China; ^2^Department of Pathogenic Biology and Immunology, School of Basic Medical Sciences, Xi'an Jiaotong University, Xi'an, China; ^3^College of Veterinary Medicine, Qingdao Agricultural University, Qingdao, China; ^4^University of Wisconsin-Madison, Madison, WI, United States

**Keywords:** reptiles, wildfowl, infectious disease, ecology, gut microbiota

The study of microbial symbioses in reptiles and wildfowl has gained increased attention due to its implications for understanding emerging infectious diseases. Reptiles and wild birds inhabit diverse ecosystems that serve as natural reservoirs for a variety of pathogens, including Adenoviridae, Circoviridae, Retroviridae, and Parvoviridae. This complex interaction has revealed both reptiles and wildfowl as potential reservoirs for emergent diseases, raising concerns about their roles in the spread of infections such as Avian Influenza Virus (AIV) and Newcastle Disease Virus (NDV) among others. The pinpointing of these viruses in their systems underlines a significant, yet underexplored, area of microbial interaction and disease dynamics.

This Research Topic aims to delve into the ecological and behavioral factors that influence disease transmission between reptiles and wildfowl, assess the health impacts on these animal populations, and identify potential transmission routes and risk factors associated with these interactions. The goal is to uncover novel aspects of pathogen transfer that could influence current practices in wildlife conservation and disease prevention.

The six studies in this Research Topic collectively address key questions: How do diet and habitat shape microbial diversity in reptiles? What roles do reptiles play as reservoirs for zoonotic pathogens? How do wildfowl contribute to the evolution and spread of viruses? Their findings not only deepen our understanding of host-microbe interactions but also highlight the need for interdisciplinary approaches to address emerging challenges.

Long-distance migratory birds play critical roles in the ecology and evolution of avian influenza viruses (AIVs) and are regarded as natural reservoirs for these viruses. Understanding the prevalence of AIVs in wildfowl is essential for effective risk assessment and preparedness against future outbreaks. Ren et al. analyzed the spatiotemporal distribution of H12 subtype AIVs worldwide and conducted a comprehensive investigation into the evolutionary and biological characteristics of an H12N2 virus isolated from a whooper swan in Central China. They concluded that the H12N2 strain is a complex reassorted virus and exhibits moderate horizontal transmission in ducks and chickens. Furthermore, the AIV strain could infect mice without prior adaptation and replicates efficiently in MDCK cells, posing a potential risk of mammalian infection. Except infection of AIVs, six types of avian astroviruses were also infected in wildfowl, which cause intestinal and other internal organ diseases. Li et al. detected 269 clinical samples for astroviruses from wild birds. The results of clinical samples showed that positive rate of DAstV-4 was more prevalent than that of DAstV-3, GoAstV-2, and GoAstV-1. Further research was needed to evaluate the pathogenicity of astrovirus in wildfowl. Deltacoronavirus was widely distributed among pigs and wild birds, and posed a significant risk of cross-species transmission. Tian Y. et al. described a novel deltacoronavirus in black-headed gull in Qinghai Lake, and closely related the HNU4-1, HNU4-2, and HNU4-3 strains originating from *Chroicocephalus ridibundus* in Yunnan province, China. Structural analysis and experiment investigated that the binding capacity of RBD of BHG-QH-2021 was similarity to human 229E strain, which indicated that the deltacoronavirus could spillover from avian to humans through recombination or mutation. These findings highlight the need to strengthen epidemiological surveillance and risk assessment of diseases originating from wildfowl.

Reptiles are ancient vertebrates, and understanding their gut microbiota can help assess their ecological health and biodiversity, providing important information about their habitat, identifying the relationship of microbial communities associated with reptile diseases. Hu et al. firstly described gut and oral microbial diversity in *Protobothrops mucrosquamatus, Elaphe dione*, and *Gloydius angusticeps*. Metagenomic analysis of fecal and oral samples revealed that three Chinese snakes had uniquely core microbial phyla comparing with other snakes. As is known, Bacteroidetes are abundant in the gut communities of many mammals but exhibit lower abundance in insectivorous mammals. However, Cong et al.'s study revealed that in addition to the three snake species mentioned above, both *Oligodon formosanus* and *Pareas chinensis* also show high Bacteroidetes abundance. This finding indicates that the role of Bacteroidetes in reptilian evolution, behavior, and digestion warrants further investigation. Like rodents, boars, bats, and birds, snakes carrying more than 34 bacterial and parasitic pathogens, which suggested that reptile were served as potential vectors of zoonosis, posing considerable disease risk to both other wildlife and humans. In lizards, Tian Z. et al. identified a PVs, named JsPV, in oral cavity of Splendid japalure. Compared to the genomic architecture of other known sauropsid PVs, JsPV, along with HfrePV1 and HfrePV2, encoded only five proteins (E7, E1, E2, L2, and L1), lacking the E6 gene—the fewest reported among lacertilian PVs, suggesting that lacertilian PVs may lack the molecular capacity to induce tumorigenesis. Phylogenetic analysis demonstrated that the lizard-derived JsPV clustered closely with gecko-associated strains (HfrePV1 and HfrePV2), forming a distinct clade separate from those associated with snakes, birds, turtles, and mammals, suggesting that these viruses may have originated from a distinct ancestral lineage in lizards and may have adapted to specific ecological niches within sympatric Lacertidae.

The studies in this Research Topic collectively highlight the complexity of microbial symbiosis and pathogen dynamics in reptiles and wildfowl. Reptilian microbiomes are tightly linked to diet and habitat, with emerging evidence of their role in zoonotic disease transmission. Wildfowl, through migration, drive viral reassortment and spread, posing risks to wildlife, livestock, and humans. Given the complex behaviors and ecological traits of wildfowl and reptiles, future research should prioritize longitudinal, large-scale investigations leveraging high-throughput sequencing to track microbial shifts over time. Expanding diagnostic tools such as multiplex PCR for rapid, on-site pathogen detection will be critical. These efforts will help map transmission networks among reptiles, wildfowl, and other species, including humans, enabling more accurate predictions of disease emergence. Ultimately, such work will deepen our understanding of how wildlife act as reservoir hosts and how their ecological behaviors shape microbial interactions and disease dynamics.

